# Quality Degradation of Chinese White Lotus Seeds Caused by Dampening during Processing and Storage: Rapid and Nondestructive Discrimination Using Near-Infrared Spectroscopy

**DOI:** 10.1155/2015/345352

**Published:** 2015-06-28

**Authors:** Lu Xu, Hai-Yan Fu, Chen-Bo Cai, Yuan-Bin She

**Affiliations:** ^1^College of Material and Chemical Engineering, Tongren University, Tongren, Guizhou 554300, China; ^2^The Modernization Engineering Technology Research Center of Ethnic Minority Medicine of Hubei Province, College of Pharmacy, South-Central University for Nationalities, Wuhan 430074, China; ^3^College of Chemistry and Life Science, Chuxiong Normal University, Chuxiong 675000, China; ^4^College of Chemical Engineering, Zhejiang University of Technology, Hangzhou 310014, China

## Abstract

Dampening during processing or storage can largely influence the quality of white lotus seeds (WLS). This paper investigated the feasibility of using near-infrared (NIR) spectroscopy and chemometrics for rapid and nondestructive discrimination of the dampened WLS. Regular (*n* = 167) and dampened (*n* = 118) WLS objects were collected from five main producing areas and NIR reflectance spectra (4000–12000 cm^−1^) were measured for bare kernels. The influence of spectral preprocessing methods, including smoothing, taking second-order derivatives (D2), and standard normal variate (SNV), on partial least squares discrimination analysis (PLSDA) was compared to select the optimal data preprocessing method. A moving-window strategy was combined with PLSDA (MWPLSDA) to select the most informative wavelength intervals for classification. Based on the selected spectral ranges, the sensitivity, specificity, and accuracy were 0.927, 0.950, and 0.937 for SNV-MWPLSDA, respectively.

## 1. Introduction

Lotus (*Nelumbo nucifera* Gaertn.) is an aquatic perennial from family Nelumbonaceae. Lotus has been cultivated for thousands of years in China and nowadays it is widely grown and common in India, Thailand, South Korea, Japan, Australia, and the US [1]. Virtually all parts of lotus, such as its seeds, rhizomes, leaves, flowers, and stamens, are consumed worldwide [2]. The most important reason for its current widespread planting is the consumption of lotus roots and seeds. Lotus seed (Lianzi in Chinese) is widely consumed as a valuable functional food in China for soups, congee, pastries, and other dishes. Recent investigations have demonstrated that lotus seeds contain many nutritional and bioactive substances, such as phospholipids, proteins, amino acids, vitamins, sugars, essential minerals [3–5], alkaloids, and flavonoids [6, 7]. Lotus seeds have also been used to treat tissue inflammation, cancer, diuretics, and some skin diseases in Ayurveda and traditional Chinese medicines [1, 8, 9]. Pharmacological experiments indicate that lotus seeds have strong antipyretic, cooling, astringent, demulcent, antioxidant properties, as well as scavenging effects on reactive nitrogen species [10–14].

There are two types of lotus seeds in China, namely, red (Honglianzi) and white (Bailianzi). Honglianzi is harvested when the seed head of the lotus is ripe or nearly ripe and Bailianzi is harvested when the seed head is still fully green, but with almost fully developed seeds. White lotus seeds (WLS) are bare kernels and are much more usually used than red lotus seeds. The quality of WLS is determined mainly by the origin, variety, ripeness, size of seeds, and the processing. Moreover, if the dried WLS has been contacted with moisture for a long time, the quality and taste of WLS can be significantly degraded. Unfortunately, lotus is mainly cultivated in a humid climate and dried WLS tends to absorb moisture in the air. Dampening can also occur during processing. Because the processing of fresh WLS can be nonuniform for all seeds in a batch, some WLS cannot be fully dried. Although producers and sellers usually will redry the seeds and recover the appearance by some physical or/and chemical processing, the taste and quality of dampened WLS can no longer be recovered. Such dampened and redried WLS should be sorted out and sold as lower-grade products. However, because the labor is expensive and it is time-consuming to manually sort out the dampened WLS, most producers will just get rid of the seriously dampened and mildewy WLS and prefer to mix and sell the regular and dampened WLS together to get a good price. Therefore, it is necessary to develop a rapid and effective method to distinguish the dampened WLS from the regular WLS.

As a promising alternative approach to the traditional analytical techniques, near-infrared (NIR) spectrometry, when combined with chemometrics, has demonstrated great potential for rapid analysis of food products [15–19]. NIR has some advantages over traditional chemical analysis, including less sample pretreatments, being fast and economical, simultaneous multicomponent characterization or analysis, and the feasibility for online analysis. Moreover, NIR is nondestructive and thus is suitable for analyzing a large number of WLS samples from supermarket shelf and small retailers.

The objective of this paper was to investigate the feasibility of using NIR spectroscopy and chemometrics to distinguish the dampened WLS objects from the regular WLS. To highlight the spectral difference caused by dampening, different data preprocessing methods were compared to select the most suitable and effective one to reduce the irrelevant spectral variations. To reduce model complexity and ensure the generalization of classification models, a moving-window technique [20] was combined with partial least squares discrimination analysis (PLSDA) [21] to select the most informative wavelength intervals for classification.

## 2. Materials and Methods

### 2.1. Collection and Labeling of Samples

Regular (167 objects) and dampened (118 objects) WLS were collected from five main producing areas in China, including Hubei (dampened/regular, 22/35 objects), Fujian (25/32 objects), Jiangsu (25/35 objects), Hunan (21/32 objects), and Zhejiang (25/33 objects). An obvious difference between the dampened and regular WLS objects is that the germ (embryo) of regular WLS will have a color of emerald green while that of dampened WLS tends to turn yellow, brown, or even black. In order to make sure that the training set includes correctly labeled samples, each WLS kernel for analysis was manually divided into two cotyledons and the embryo of the kernel was examined. All the kernels were dried fully in the sun before spectrometry analysis.

### 2.2. NIR Measurement

The NIR spectra were collected with the bare kernels in the diffuse reflectance mode using a Bruker TENSOR 37 FTIR spectrometer (Bruker Optics, Ettlingen, Germany). A fiber bundle was used to illuminate the sample and collect the scattered light. The fiber probe was placed to contact directly with equatorial region of a kernel. Considering the differences in the internal composition of a kernel, the diffuse reflectance spectrum was obtained by averaging the three measurements around the kernel. Each spectrum was the average of 64 scans, and more scans did not reduce the signal noise significantly. The range of the raw spectra was from 12,000 to 4000 cm^−1^, and the data were measured with an interval of 1.929 cm^−1^ and a resolution of 4 cm^−1^, so each raw spectrum has 4148 individual data points. The work temperature was kept at 25°C and the sequence of NIR analysis for all the objects was randomly arranged.

### 2.3. Preprocessing, Outliers Diagnosis, and Data Splitting

All the data preprocessing, modeling, and analysis were performed on MATLAB 7.0.1 (Mathworks, Sherborn, MA). Smoothing using the Savitzky and Golay (S-G) algorithm of polynomial fitting [22] was used to reduce the random noise present in the raw spectra. Taking derivatives can enhance spectral resolution and remove linear baseline shifts, so second-order derivative (D2) spectra were also used. D2 spectra were also computed using the S-G algorithm to avoid the degradation of signal-to-noise ratio (SNR) caused by direct differencing. Considering the rough surfaces of WLS kernels, standard normal variate (SNV) [23] was performed to reduce the influence of scattering and path variations.

For outlier diagnosis, the Stahel-Donoho estimate (SDE) of outlyingness [24] was performed on the raw spectra. By repeatedly projecting each high-dimensional object onto randomly generated unit vectors for many times, SDE enables observing the distribution of high-dimensional data in a low-dimensional space. Based on the robust location (median) and scatter estimator (median absolute deviation, MAD), the SDE outlyingness value for each object can be computed and tested according to a normal distribution. The number of random projections was 1000 in this work.

After removal of outliers, the DUPLEX algorithm [25] was used to divide the measured objects into a representative training set and test set. DUPLEX firstly selects the two farthest samples and puts them in the training set based on Euclidean distance; then it selects the two currently farthest samples and puts them in the test set. The above procedure is repeated until one has obtained sufficient objects for test and the remaining objects are all put in the training set. By alternatively selecting the farthest objects for the training set and test set, DUPLEX can obtain a training set and test set that are evenly spread as much as possible over the whole experimental region. Because the distributions of regular and dampened WLS were heterogeneous, DUPLEX algorithm was performed separately on the regular and dampened objects.

### 2.4. PLSDA and Moving-Window PLSDA (MWPLSDA)

PLSDA is a classification method based on partial least squares (PLS) regression. As a key method of chemometrics, PLS has been successfully applied to various regression and calibration problems. For two-class classification, supposing an *n* × *p* matrix **X** including *p* wavelength variables for *n* training objects, a dummy response vector **y** (*n* × 1) is constructed to match with each object in **X**; for example, +1 and −1 were used to denote regular and dampened WLS samples, respectively. Therefore, the critical value of predicted response values was set to be 0; namely, an object with a predicted response value above/under 0 would be assigned to the regular/dampened class.

The motivation for moving-window technique is the continuity of spectral responses; for example, for NIR spectra, the spectral bands are caused by vibration and turning of molecules. The continuity of spectral responses means if a single wavelength carries useful information for classification, so does the spectral interval containing its neighboring wavelengths. For PLSDA, the spectral intervals with small uncertainty can be ascertained by the low misclassification rate and model complexity. In MWPLSDA, a spectral window with a given width is moving along the total wavelength range. Each spectral window is used to build a PLSDA model to classify the regular and dampened objects; the training errors of each PLSDA model with different model complexity or numbers of latent variables (LVs) are then plotted against the windows (wavelengths). From the above plot, spectral intervals highly informative for classification can be identified by less model complexity and lower training errors. Finally, all selected intervals are combined to develop a PLSDA model for classification. The model complexity of MWPLSDA was estimated by Monte Carlo cross validation (MCCV) [26].

Sensitivity and specificity [27] were used to compare the performance of different classification models and data preprocessing methods. Denoting regular WLS as “positives” and dampened WLS as “negatives,” sensitivity (Sens) and specificity (Spec) were computed as

(1) Sens=TPTP+FN,Spec=TNTN+FP,

where TP, FN, TN, and FP denote the numbers of true positives, false negatives, true negatives, and false positives, respectively.

The overall accuracy (Accu) of classification was also used:

(2) $$\begin{array}{c} Accu=\frac{TN+TP}{TN+TP+FN+FP}. \end{array}$$

## 3. Results and Discussions

**Figure 1:**
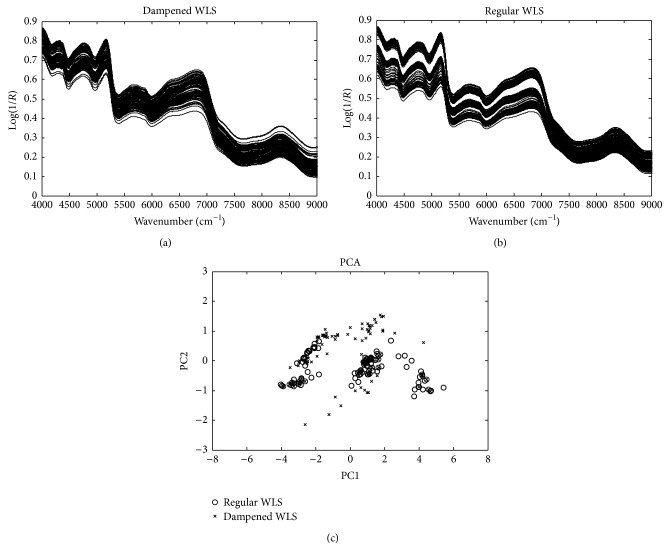
The raw NIR spectra and principal component analysis of 167 regular and 118 dampened WLS objects.

Because the spectral range of 9000–12000 cm^−1^ was seriously influenced by baseline shifts, only the spectral range of 4000–9000 cm^−1^ was used for chemometric analysis. The raw NIR spectra of the 118 dampened and 167 regular WLS objects were plotted in Figure 1. Seen from Figure 1, the spectra of dampened and regular WLS have very similar absorbance bands. For the raw spectra, the regular WLS objects demonstrated more spectral variations among different producing areas than the dampened objects. Principal component analysis (PCA) was performed on the raw data (4000–9000 cm^−1^) to demonstrate the distribution of dampened and regular samples. The first two principal components (PCs) explained 93.19% of the total data variances. Seen from the PCA plot, the regular and dampened WLS overlapped seriously in the space spanned by the first two PCs, indicating that wavelength selection was required to find the informative wavelengths for accurate classification of the two classes.

**Figure 2:**
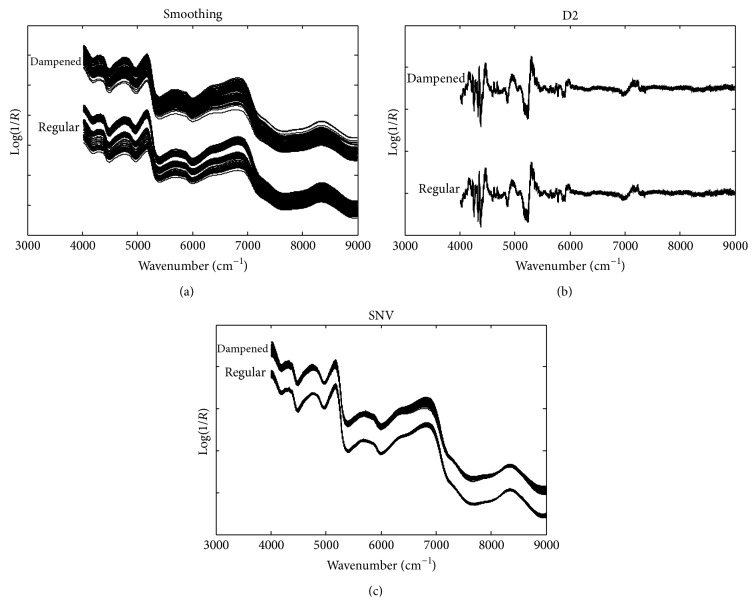
The NIR spectra of regular and dampened WLS preprocessed by smoothing, taking second-order derivatives (D2) and standard normal variate (SNV).

Figure 2 demonstrates the preprocessed spectra of dampened and regular WLS. An extra shift of Log(1/*R*) was included to distinguish the regular and dampened WLS. Compared with the raw spectra, although spectral smoothing can slightly enhance the SNR, the baseline shifts in the raw spectra were not removed. By taking D2 spectra, the baseline shifts were largely reduced and much detailed and high-frequency information was obtained. By comparison of the D2 spectra, the regular and dampened WLS had differences in the relative intensities of some peaks, but it was still difficult to distinguish the two classes by the naked eye. SNV transformation removed much of the unwanted variations in the two classes. The actual effects of data preprocessing should be evaluated in terms of classification performance.

Diagnosis of outliers was performed based on the raw spectra (4000–9000 cm^−1^). With 1000 random projections, the SDE outlyingness values for the 167 regular and 118 dampened WLS were computed. According to the experiential 3-*σ* rule, any objects with an outlyingness value above 3 would be detected as outliers. For both regular and dampened objects, no outliers were detected and removed because all the outlyingness values were significantly less than 3. For data splitting, the DUPLEX method was performed on the raw spectra of regular and dampened WLS separately. Each class was divided into training and testing objects, which were then combined to form the final training and test sets. Therefore, the final training set had 190 objects (112 regular and 78 dampened objects) and 95 test samples (55 regular and 40 dampened objects).

With different preprocessing methods, full-spectrum (4000–9000 cm^−1^) PLSDA and MWPLSDA were developed. For both full-spectrum PLSDA and MWPLSDA, the model complexity was estimated by MCCV. In this work, the number of MCCV random splittings was 100 and considering the size of the training set, each time 30% of the training objects were left out for prediction. The overall misclassification rate of MCCV was computed with different latent variables (LVs) and an economical classification model was selected so that including more LVs could not significantly reduce the misclassification rate.

**Figure 3:**
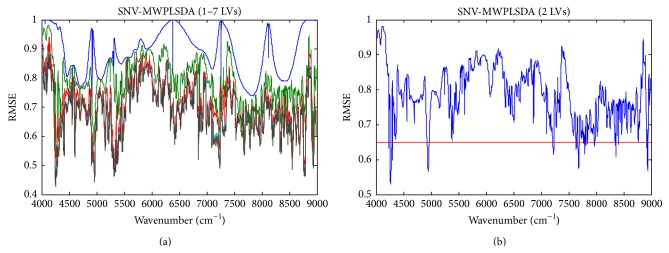
The RMSE by SNV-MWPLSDA with different latent variables.

**Figure 4:**
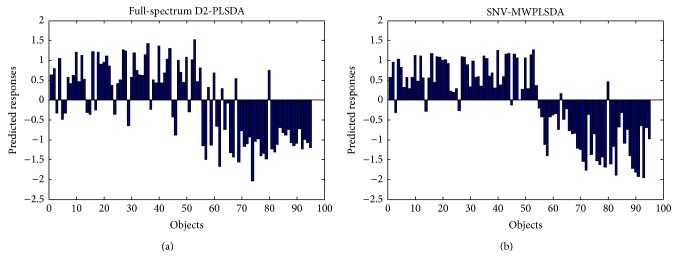
The prediction results of full-spectrum D2-PLSDA and SNV-MWPLSDA for 55 regular WLS (objects 1–55) and 40 dampened WLS (objects 56–95).

For MWPLSDA, the moving window contained 19 wavelengths. For wavelength selection, the root mean squared error (RMSE) of the dummy response variable by each window model was plotted against the location (center wavelength) of the window.  Figure 3 demonstrates the RMSE of MWPLSDA with different LVs. Seen from Figure 3, the RMSE obtained with 2 LVs was informative to select the useful spectral intervals for classification because including more LVs could not reduce the RMSE significantly. To find proper spectral intervals, with 2 LVs, the spectral intervals with RMSE lower than 0.65 were selected. Moreover, because PLSDA based on separate wavelengths tends to be more instable than those based on continuous wavelength intervals, only the continuous wavelength intervals were used to build the final MWPLSDA models. The results by full-spectrum PLSDA and MWPLSDA with different preprocessing methods are shown in Table 1. Seen from Table 1, with full spectrum, the classification accuracy of PLSDA was unsatisfactory. Although D2 and SNV could improve the classification, there were still a considerable part of objects that were wrongly predicted. The best classification accuracy by full-spectrum PLSDA was 0.821 obtained with D2 preprocessing. The classification accuracy was largely improved by MWPLSDA with the raw spectra and all the three preprocessing methods. The best classification performance was obtained by SNV-MWPLSDA, with sensitivity, specificity, and accuracy of 0.927, 0.950, and 0.937, respectively. The prediction results of SNV-MWPLSDA are shown in Figure 4. The results indicate that MWPLSDA was effective in selecting the relevant wavelengths for distinguishing dampened and regular WLS objects.

## 4. Conclusion

Rapid and nondestructive discrimination of dampened and regular WLS was developed using NIR spectroscopy and chemometrics. With MWPLSDA, informative spectral intervals were selected and the classification performance was largely improved. By comparison of the full-spectrum PLSDA and MWPLSDA, wavelength selection played a more important role than spectral preprocessing, indicating that the NIR analysis was well designed and controlled. Although it is difficult to exhaustively collect the WLS objects from all the different producing areas, the classification of regular/dampened WLS from main producing areas was reliable and accurate. The model complexity of all PLSDA or MWPLSDA was low (with 2–4 LVs), indicating the good generalization performance of the classification models. Therefore, it is feasible to generalize the models by including more WLS objects for training. Our future work will be focused on NIR analysis of the bioactive components influenced by storage and dampening.

## Figures and Tables

**Table 1:** The model parameters and prediction results of full-spectrum PLSDA and MWPLSDA models.

Preprocessing	Models	Spectral range (cm^−1^)	LVs^a^	Sensitivity^b^	Specificity^c^	Accuracy^d^
Raw data	PLSDA	4000–9000	4	0.727 (40/55)	0.775 (31/40)	0.747 (71/95)
Smoothing	PLSDA	4000–9000	4	0.727 (40/55)	0.800 (32/40)	0.758 (72/95)
D2	PLSDA	4000–9000	3	0.782 (43/55)	0.875 (35/40)	0.821 (78/95)
SNV	PLSDA	4000–9000	3	0.800 (44/55)	0.775 (31/40)	0.789 (75/95)
Raw data	MWPLSDA	4904–4991, 4746–4811	3	0.855 (47/55)	0.850 (34/40)	0.853 (81/95)
Smoothing	MWPLSDA	4904–4991, 4746–4811	3	0.855 (47/55)	0.850 (34/40)	0.853 (81/95)
D2	MWPLSDA	7054–7129, 5444–5494, 5234–5348	2	0.873 (48/55)	0.95 (38/40)	0.905 (86/95)
SNV	MWPLSDA	4236–4300, 4908–4979, 7191–7243, 7608–7708, 7943–7992, 8911–8956	3	0.927 (51/55)	0.950 (38/40)	0.937 (89/95)

^a^Number of PLSDA latent variables.

^b^The numbers in the brackets indicate TP/(TP + FN).

^c^The numbers in the brackets indicate TN/(TN + FP).

^d^The numbers in the brackets indicate (TN + TP)/(TN + TP + FP + FN).
